# The TLR7 agonist imiquimod induces anti-cancer effects via autophagic cell death and enhances anti-tumoral and systemic immunity during radiotherapy for melanoma

**DOI:** 10.18632/oncotarget.15326

**Published:** 2017-02-15

**Authors:** Jeong Hyun Cho, Hyo-Ji Lee, Hyun-Jeong Ko, Byung-Il Yoon, Jongseon Choe, Keun-Cheol Kim, Tae-Wook Hahn, Jeong A. Han, Sun Shim Choi, Young Mee Jung, Kee-Ho Lee, Yun-Sil Lee, Yu-Jin Jung

**Affiliations:** ^1^ Department of Biological Sciences, Kangwon National University, Chuncheon, Republic of Korea; ^2^ College of Pharmacy, Kangwon National University, Chuncheon, Republic of Korea; ^3^ Department of Veterinary Medicine, Kangwon National University, Chuncheon, Republic of Korea; ^4^ Department of Microbiology, School of Medicine, Kangwon National University, Chuncheon, Republic of Korea; ^5^ Department of Biochemistry and Molecular Biology, School of Medicine, Kangwon National University, Chuncheon, Republic of Korea; ^6^ Department of Medical Biotechnology, College of Biomedical Science, Kangwon National University, Chuncheon, Republic of Korea; ^7^ Department of Chemistry, Kangwon National University, Chuncheon, Republic of Korea; ^8^ Division of Radiation Cancer Research, Korea Institute of Radiological and Medical Sciences, Nowon-gu, Seoul, Republic of Korea; ^9^ Graduate School of Pharmaceutical Sciences, Ewha Womans University, Seodaemun-gu, Seoul, Republic of Korea

**Keywords:** melanoma, TLR7, imiquimod (IMQ), autophagy, radiotherapy

## Abstract

Toll-like receptor (TLR) ligands are strongly considered immune-adjuvants for cancer immunotherapy and have been shown to exert direct anti-cancer effects. This study was performed to evaluate the synergistic anti-cancer and anti-metastatic effects of the TLR7 agonist imiquimod (IMQ) during radiotherapy for melanoma. The pretreatment of B16F10 or B16F1 cells with IMQ combined with γ-ionizing radiation (IR) led to enhanced cell death via autophagy, as demonstrated by increased expression levels of autophagy-related genes, and an increased number of autophagosomes in both cell lines. The results also confirmed that the autophagy process was accelerated via the reactive oxygen species (ROS)-mediated MAPK and NF-κB signaling pathway in the cells pretreated with IMQ combined with IR. Mice subcutaneously injected with melanoma cells showed a reduced tumor growth rate after treatment with IMQ and IR. Treatment with 3-methyladenine (3-MA), ameliorated the anti-cancer effect of IMQ combined with IR. Additionally, the combination therapy enhanced anti-cancer immunity, as demonstrated by an increased number of CD8^+^ T cells and decreased numbers of regulatory T cells (Treg) and myeloid-derived suppressor cells (MDSCs) in the tumor lesions. Moreover, the combination therapy decreased the number of metastatic nodules in the lungs of mice that were injected with B16F10 cells via the tail vein. In addition, the combination therapy enhanced systemic anti-cancer immunity by increasing the abundances of T cell populations expressing IFN-γ and TNF-α. Therefore, these findings suggest that IMQ could serve as a radiosensitizer and immune booster during radiotherapy for melanoma patients.

## INTRODUCTION

Toll-like receptors (TLRs) are known to recognize pathogen associated molecular patterns (PAMPs) to initiate innate immunity and to induce adaptive immune reactions [1, 2]. Synthetic TLR agonists are regarded as potential candidate drugs for development as therapeutic agents for infectious diseases, autoimmune diseases, and even cancers [3, 4]. Although the anti-tumoral activities of TLR ligands have previously been demonstrated in a variety of cancer cell types, the primary role of TLR ligands was identified as an indirect enhancement of the immune response activated by dendritic cells (DCs) [5, 6]. Imiquimod (IMQ), a synthetic TLR7 agonist, has been reported to induce apoptosis via modulation of the expression of Bcl-2/Bax in cells of various cancer types, including renal cell carcinoma [7], intraepithelial neoplasia [8], squamous cell carcinoma [9] and basal cell carcinoma [10]. Moreover, the inhibition of caspase activation prevents the IMQ-induced apoptosis of tumor cells [11]. These results indicate that IMQ exerts direct anti-tumoral effects via activation of the TLR7 signaling pathway. We previously reported that IMQ induced the death of colon cancer cells via autophagy [12] and sensitized breast cancer cells to radiotherapy [13]. Moreover, it has been reported that poly(I:C), a TLR3 agonist, has direct anti-tumoral activity that is induced by autophagy in melanoma [14]. However, the cellular mechanisms underlying the TLR-induced anti-cancer activities have not been fully elucidated to date.

TLR signaling has also been linked to autophagy in antigen-presenting cells, and the activation of this process results in an accelerated elimination of intracellular pathogens as an innate immune defense mechanism [15]. Autophagy is involved in not only the eradication of pathogens by the immune system but also the maintenance of the homeostatic clearance of damaged organelles or cytosolic proteins [16]. During autophagy, cells exhibit extensive internal membrane remodeling, including the engulfment of portions of the cytoplasm in large double-membrane vesicles. These autophagosomes dock and fuse with lysosomes, and the contents of the fused vacuoles are ultimately degraded. A group of genes known as ATG genes, which are conserved from yeast to humans, has been found to control autophagy [17]. Programmed cell death is an important target of traditional cancer treatment. Apoptosis, the most thoroughly studied form of programmed cell death, is characterized by a primary reliance on caspases [18]. Another programmed cell death mechanism that is clearly distinct from apoptosis is autophagy. Apoptosis and autophagy have been shown to function in tumor suppression [19].

It has been shown that cancer can develop under specific environments generated by chronic inflammation [20]. Cancer cells undergo intrinsic genetic changes and are surrounded by an inflammatory state that directly influences their growth and spread. This prolonged condition also generates an immuno-suppressive environment by recruiting suppressor cells, including CD4^+^CD25^+^Foxp3^+^ regulatory T cells (Treg) [21], myeloid-derived suppressor cells (MDSCs) [22, 23], tumor-associated macrophages (TAMs) [24], N2-polarized neutrophils [25], and regulatory/tolerogenic DCs [26]. Additionally, several mediators such as TGF-β, IL-10 [27] and indoleamine-2,3-dioxygenase [28] enable the escape of the cancer cells from immune surveillance [29]. Therefore, trials of interventions aiming to enhance immunity via cancer-specific and microenvironmental immuno-suppressive mechanisms have suggested that these interventions improve the clinical outcome of cancer [30].

This study evaluated the synergistic anti-cancer and anti-metastatic effects of IMQ treatment during radiotherapy for melanoma. Treatment with IMQ induced cell death in the mouse melanoma cell lines B16F1 and B16F10 via autophagy. In addition, IMQ combined with IR enhanced tumor regression in an *in vivo* mouse model and increased the survival duration of a metastatic model, and these findings agree with the results of enhanced anti-cancer immunity in local tumor lesions and in the circulation of tumor-bearing mice. Therefore, the results indicate that IMQ could be developed as a synergistic adjuvant to cancer radiotherapy for melanoma patients.

## RESULTS

### IMQ treatment increases the autophagic death of melanoma cells during radiotherapy

**Figure 1:**
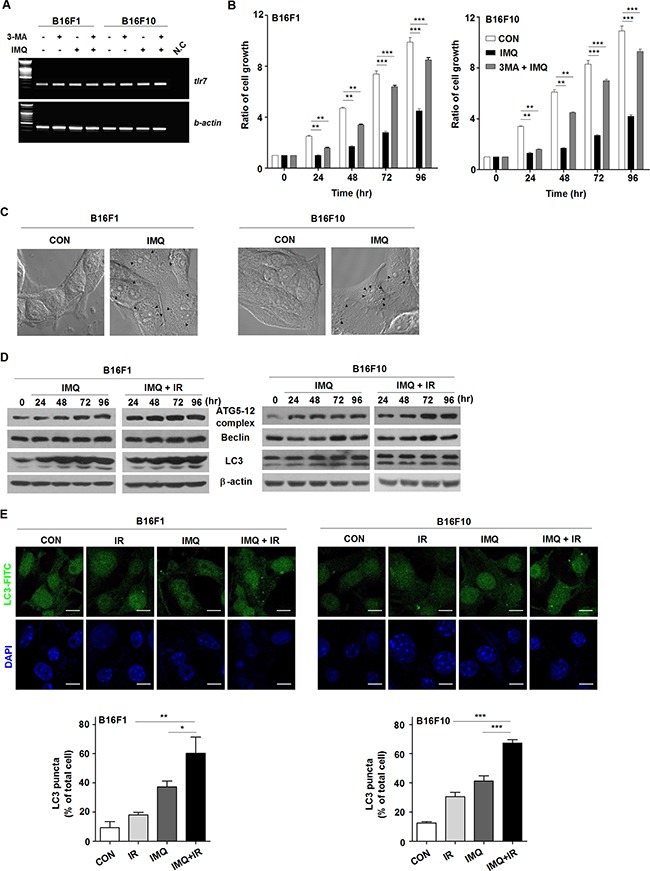
IMQ combined with IR enhance the autophagic death of melanoma cells **A**. The expression of TLR7 in the B16F1 and B16F10 melanoma cell lines was detected. β-actin was used as an internal standard. **B**. The growth rates of untreated cells (CON), IMQ-treated cells (IMQ), and 3-MA and IMQ-treated cells (3MA+IMQ) of both cell lines were measured. **C**. Autophagosome vacuoles detected in IMQ-treated cells via microscopy. **D**. The expression levels of autophagy-related genes in IMQ-treated or IMQ+IR-treated B16F1 or B16F10 cells were detected by western blot analysis. **E**. The LC3-positive B16F1 and B16F10 cells after treatment with IMQ alone or in combination with IR were detected via immunofluorescent staining. The number of cells containing LC3-positive puncta was counted under a microscope, and the percentage of cells containing LC3-positive puncta relative to the total cell number was calculated. The mean percentages ± SE from triplicate measurements are shown. All of the experiments were independently repeated three times for each condition with similar results. Significant differences are indicated by *p < 0.05, **p < 0.01, and ***p < 0.001.

In a previous study, we found that IMQ induced the autophagic death of not only colon cells [12] but also radioresistant MCF-7 breast cancer cells [13]. To confirm that IMQ acts as a potent radiosensitizer against melanoma by enhancing autophagic cell death, TLR7 expression was first confirmed in B16F1 and B16F10 cell lines via RT-PCR analysis. The results showed that both melanoma cell lines expressed the TLR7 transcript and that the level of TLR7 expression in the cells was not changed by treatment with IMQ alone or IMQ and 3-MA, an autophagy inhibitor (Figure 1A). Twenty-four hours after treatment with IMQ, a significantly reduced growth rate was observed in both cell lines (Figure 1B). Treatment with 3-MA resorted the survival rate of the IMQ-treated cells to a level that was similar to that of the control cells. Moreover, several autophagic vesicles were detected under phase-contrast microscopy when cells were treated with IMQ for 24 h (Figure 1C). These results combined with the evidence that cell growth was not inhibited by IMQ treatment in the cells in which Myd88 was knocked down confirm that this cell death is dependent on TLR7 (Supplementary Figure 1).

Given that melanoma has been established to be resistant to radiation-induced cell death [32], we investigated whether IMQ enhances the radiosensitivity of melanoma cells via autophagy-induced cell death. IR treatment accelerated the reduction in the survival rate of cells treated with IMQ compared with cells that were not exposed to IR (data not shown). Because the conversion of microtubule-associated protein 1 light chain (LC3) to LC3-II is a crucial molecular event in autophagy, LC3-II expression in melanoma cells after incubation with IMQ alone or IMQ combined IR was evaluated. During autophagy, LC3 is processed to soluble LC3-I, and LC3-I is in turn modified to membrane-bound LC3-II [31]. The mobilization shift from LC3-I to LC3-II was detected in B16F1 and B16F10 cells starting after 24 h to 48 h of incubation with IMQ and strongly expressed LC3-II was intensified in IR-exposed cells pretreated with IMQ (Figure 1D). Moreover, the finding of significantly increased expression levels of the Atg5-12 complex and beclin-1 beginning after 24 h of incubation provided additional strong evidence supporting the hypothesis that IMQ combined with IR induced autophagy in mouse melanoma cells (Figure 1D). To confirm that pretreatment with IMQ combined with IR accelerated the formation of autophagosomes endogenously expressing LC3, immunofluorescent staining was performed (Figure 1E). Because LC3 specifically binds to forming autophagosomes, the number of endogenous LC3-positive vesicles reflects the extent of autophagosome formation. As shown in Figure 1E, LC3 aggregates were formed in B16F1 and B16F10 cells treated with IMQ or IR alone. However, the number of autophagosomes was increased by up to 1.5-fold in cells pretreated with IMQ for 3 h and exposed to IR compared with the number detected in cells treated with IMQ alone, and this finding was obtained for both cell lines. Therefore, we speculated that treatment with IMQ sensitizes mouse melanomas cells to IR exposure by stimulating the autophagic cell death machinery.

### IMQ treatment combined with IR enhances autophagy via the ROS-mediated ERK signaling pathway in melanoma cells

**Figure 2:**
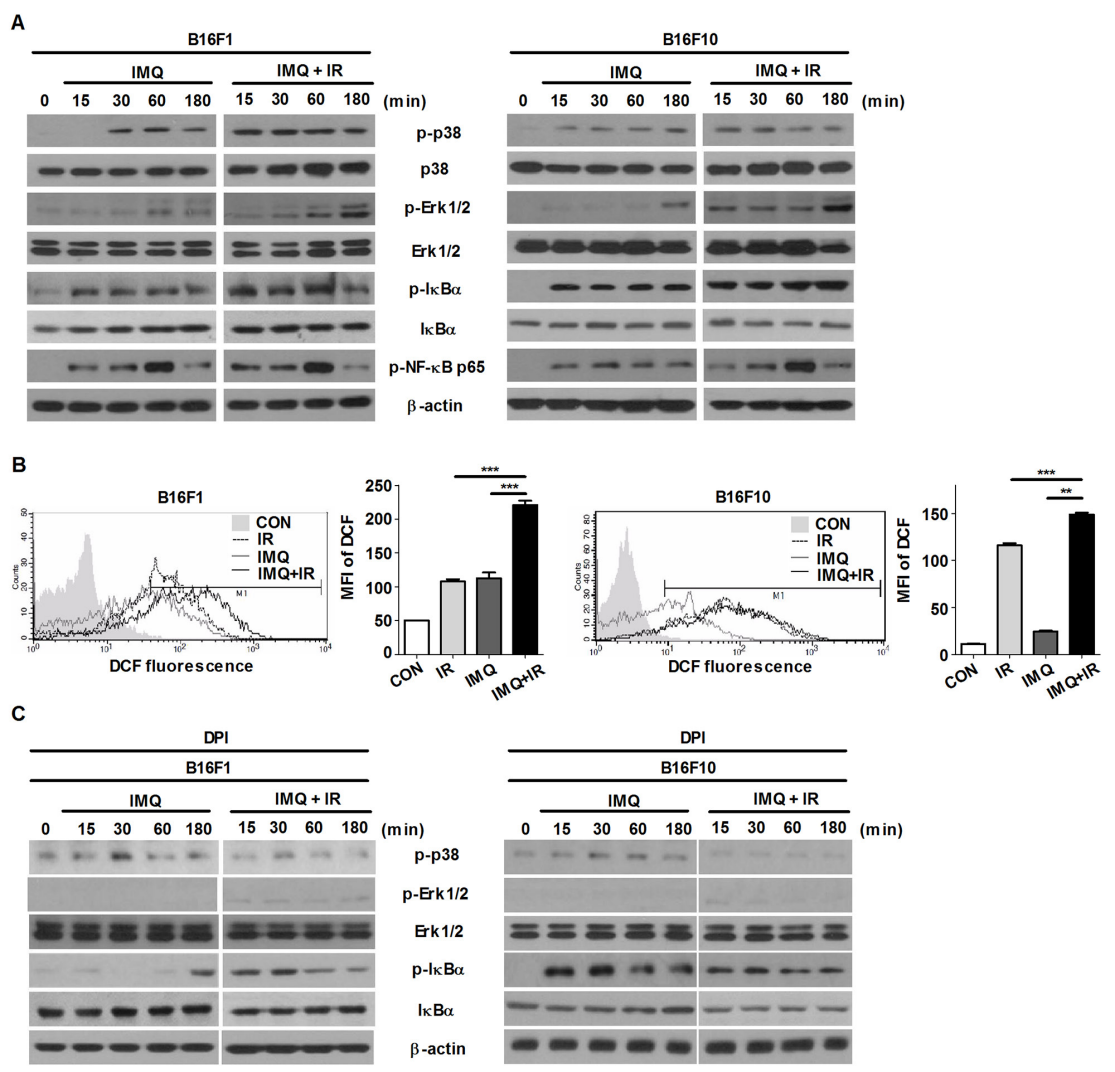

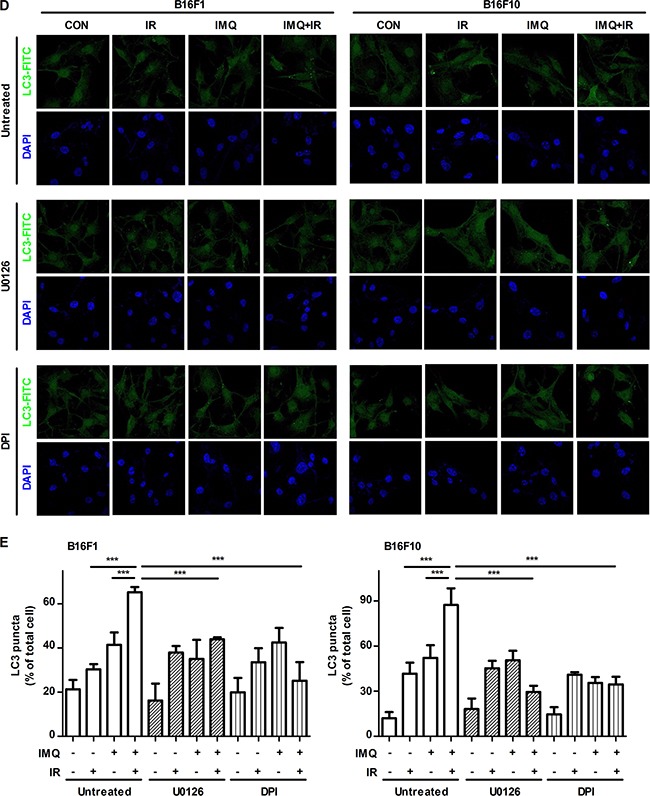
IMQ treatment combined with IR accelerates the activation of the ROS-mediated ERK signaling pathway **A**. The changes in the expression of MAPK and NF-κB signaling molecules in B16F1 and B16F10 cells treated with IMQ (IMQ) or IMQ combined with IR (IMQ+IR) were determined through western blot analysis. **B**. B16F1 and B16F10 cells were labeled with 5-[and-6]-chloromethyl-2′, 7′-dichlorodihydro fluorescein diacetate (DCFH-DA) and then evaluated using flow cytometry. The following groups were included in the analysis: untreated cells (CON), IMQ-treated cells (IMQ), IR-treated cells (IR) and cells treated with IMQ combined with IR (IMQ+IR). The mean value ± standard deviation from triplicate measurements are shown. The MFI refers to the mean fluorescence intensity. Significant differences are indicated by **p < 0.01, and ***p < 0.001. **C**. B16F1 and B16F10 cells were treated with IMQ (IMQ) or IMQ combined with IR (IMQ+IR) in the presence or absence of ROS scavenger, DPI. The changes in the expression of MAPK and NF-κB signaling molecules were determined through western blot analysis. **D**. The LC3-positive B16F10 or B16F10 cells after treatment with IMQ or IR alone or with IMQ+IR with or without U0126 or DPI were detected via immunofluorescent staining. **E**. The number of cells containing LC3-positive puncta was counted under a microscope, and the percentage of cells containing LC3-positive puncta relative to the total cell number was calculated. The mean percentages ± SE from triplicate measurements are shown. Significant differences are indicated by *p < 0.05, **p < 0.01, and ***p < 0.001.

Several recent studies have indicated that ROS-mediated activation of the MAPK signaling pathway increase the autophagy in various cancer cells [33–37]. Oxidative stress is caused by excessive reactive oxygen species (ROS), which play important roles in the induction of cell death after radiation treatment [38, 39]. To verify the signaling pathway involved in the acceleration of autophagic cell death in melanoma after treatment with IMQ combined with IR, the expression level of genes in the MAPK and NF-κB pathway were examined (Figure 2A). The phosphorylated forms of ERK1/2 in B16F10 cells were elevated starting after 15 min of incubation with IMQ and were rapidly accumulated after treatment with both IMQ and IR. In addition, the level of phosphorylated p38 was also highly increased in the cells treated with both IMQ and IR. However, activation of the NF-κB signaling pathway was not highly activated in the cells treated with IMQ combined with IR compared with the cells treated with IMQ alone.

Moreover, the results revealed that ROS production was substantially increased in the cells pretreated with IMQ for 3 h and exposed to IR compared with that observed in the cells treated with IMQ or IR alone and this finding was obtained for both cell lines (Figure 2B). However, the expression levels of phosphorylated ERK1/2 and p38 were diminished when melanoma cells were pretreated with ROS scavenging agent, diphenyleneiodonium chloride (DPI) (Figure 2C). To confirm that inhibition of ERK signaling pathway or elimination of ROS production abolish autophagy that is induced by the treatment of IMQ combined with IR, immunofluorescent staining was performed to detect endogenously aggregation of LC3. As shown in Figure 2D and 2E, the MEK inhibition or the scavenging ROS markedly reduced LC3 aggregation in both cells. Therefore, these results indicated that IMQ treatment activated autophagic cell death via the ROS-mediated ERK signaling pathway and that the cell death machinery could be markedly accelerated in melanoma cells treated with IMQ combined with IR.

### IMQ treatment sensitizes melanoma to radiotherapy

**Figure 3:**
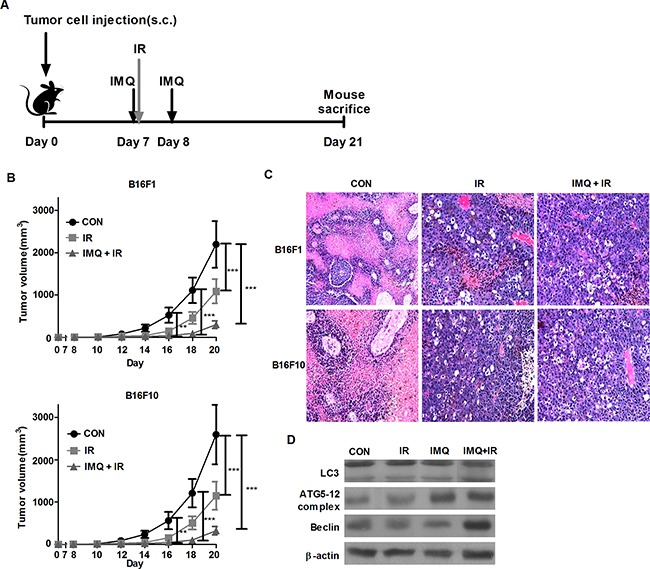
IMQ radiosensitizes melanoma in an *in vivo* mouse model **A**. Experimental schedule of tumor cell injection, IMQ treatment and IR exposure. **B**. C57BL/6 mice (n=4/group) were treated with saline (CON), IR alone (IR) or IMQ combined with IR (IMQ+IR) after inoculation with 5 × 10^5^ B16F1 or B16F10 cells. The tumor volume was measured every day using a skin caliper. All of the experiments were independently repeated three times for each condition with similar results. Significant differences are indicated by **p < 0.01 and, ***p < 0.001. **C**. The histological analysis of the tumor lesions of each group was performed 21 days after tumor cell injection. **D**. Proteins were extracted from each tumor on day 21 and then subjected to western blot analysis.

To investigate whether IMQ exerts a synergistic anti-cancer effect with IR in an *in vivo* mouse model, B16F1 or B16F10 tumor-bearing C57BL/6 mice were used (Figure 3A). First, 5 × 10^5^ melanoma cells were injected into the flanks of mice, and tumor growth was then measured at the indicated time points over a period of 21 days (Figure 3B). The tumor growth rate was significantly suppressed in the IR-treated group, and this effect was enhanced in the B16F1 or B16F10 tumor-bearing mice pre-treated with IMQ for 6 hours prior to IR exposure on day 7 and then administered another dose of IMQ on day 8. A histological analysis of tumor sections clearly showed vacuolar structures in melanoma cells treated with IMQ combined with IR. This finding demonstrates that autophagic cell death occurred in the tumor foci (Figure 3C). Moreover, at the end of the 21-day period, the expression levels of the autophagy-related molecules LC3, Atg5-12 complex, and Beclin-1 were significantly higher in tumor tissues from mice subjected to IMQ treatment combined with IR exposure (Figure 3D).

### Inhibition of autophagy abolishes the anti-cancer effect of IMQ

**Figure 4:**
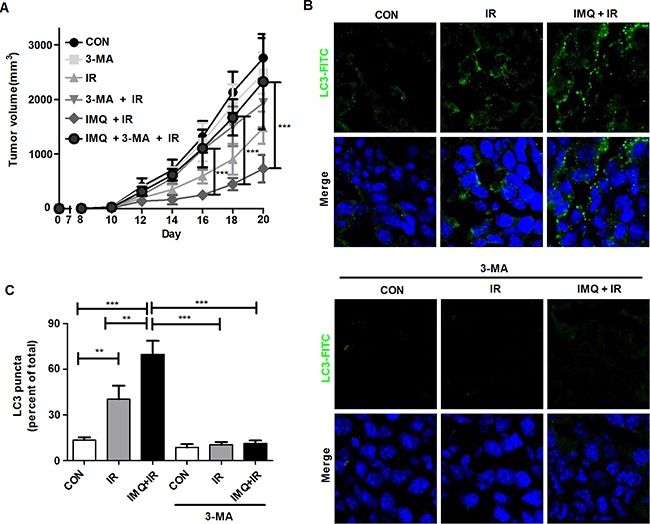
3-MA treatment restores the tumor growth rate in melanoma-bearing mice **A**. The tumor volumes in C57BL/6 mice (n=6/group) treated with saline (CON), 3-MA (3-MA), IR alone (IR) IR followed by 3-MA (IR+3-MA), IMQ combined with IR (IMQ+IR) or IMQ combined with IR and 3-MA (IMQ+IR+3-MA) were measured. Significant differences are indicated by **p < 0.01, and ***p < 0.001. **B**. Endogenous LC-3 expression in tumor tissue from six groups of tumors was determined via immunofluorescent staining. The tissues were stained with DAPI to visualize nuclei (blue) and were immunolabeled with an anti-LC3 antibody, which was detected via the addition of FITC-conjugated IgG (green). **C**. The number of cells containing LC3-positive puncta was counted under a microscope, and the percentage of cells containing LC3-positive puncta relative to the total cell number was calculated. The mean percentages ± SE from triplicate measurements are shown. Significant differences are indicated by **p < 0.01, and ***p < 0.001.

Although a synergistic anti-cancer effect was detected in the group of mice treated with IMQ combined with IR, it was unclear whether the simultaneous administration of IMQ and IR accelerated autophagic cell death in a mouse model. In these experiments, the mice were subcutaneously treated with 3-MA every day for seven days after IR exposure, and tumor growth was measured (Figure 4A). Treatment with 3-MA restored the tumor volume in the B16F10-inoculated mice to the volume detected in the control mice. The tumor volume in the 3-MA-injected mice was more than 5.8-fold greater than that found in the non-injected mice treated with IMQ and IR on day 16; furthermore, this difference in tumor volume between the two groups of mice continued to increase during the 21-day period. As shown in Figure 4B and 4C, LC3 aggregates were formed in the tumors that were treated with both IMQ and IR, and LC3 expression was significantly decreased in the 3-MA-treated groups administered IMQ either alone or in combination with IR. Therefore, IMQ in combination with IR synergistically induced cell death via autophagy in melanoma cells.

### IMQ treatment induces anti-cancer immunity in tumor lesions

**Figure 5:**
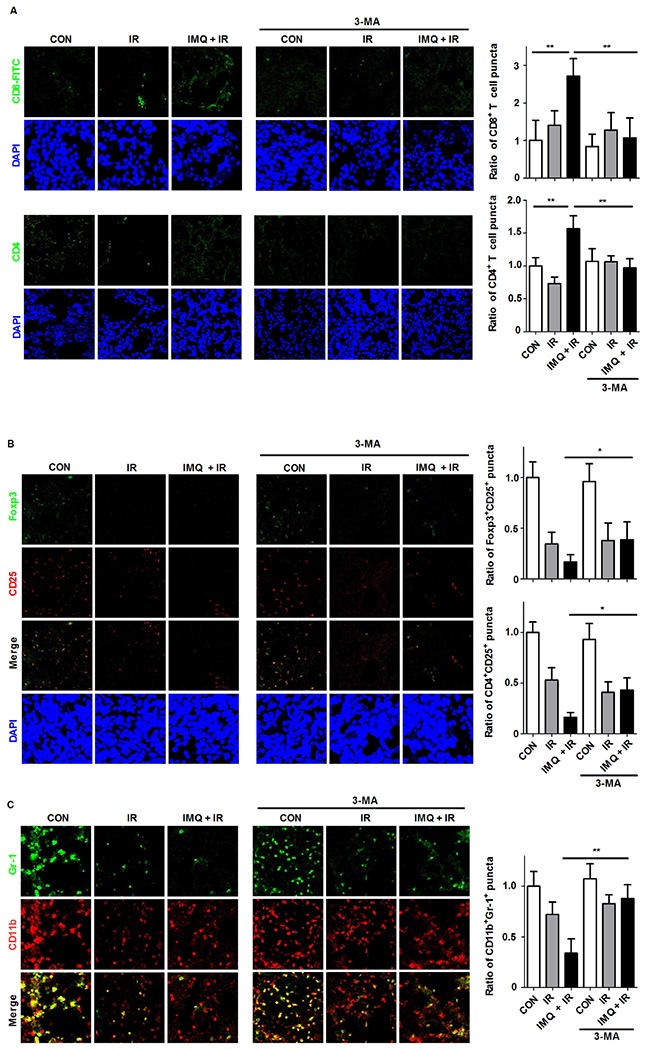
IMQ combined with IR enhances lymphocyte infiltration but decreases MDSC abundance in tumor lesions Immunostaining was performed for each tumor section 21 days after B16F10 cell inoculation. **A**. Infiltrated T cells were observed in the frozen tumor sections. The tissues were stained with DAPI to visualize nuclei (blue) and were immunolabeled with anti-CD4 and anti-CD8 antibodies, which were detected via the addition of FITC-conjugated IgG (green). The numbers of CD8^+^ and CD4^+^ T cell puncta were determined under a confocal microscope and are presented as proportions relative to the number of corresponding cells in the control group. **B**. The Treg population was detected with anti-CD25 (PE-conjugated, red) and anti-Foxp3 (FITC-conjugated, green) antibodies. The Treg numbers were counted in merged images captured via confocal microscopy. **C**. The MDSC population was detected using anti-CD11b (PE-conjugated, red) and anti-Gr1 (FITC-conjugated, green) antibodies. For all the experiments, the mean values ± SE from eight measurements are shown. Significant differences are indicated by *p < 0.05, **p < 0.01, and ***p < 0.001.

Melanoma has been reported to serve as an immunogenic cancer; therefore, the infiltration of human melanoma tissues with lymphocytes has been shown to correlate with improved clinical outcome [40]. To verify that anti-cancer immunity was enhanced in melanoma lesions upon treatment with IMQ and IR, the frequencies of the infiltrating lymphocytes and MDSC populations were measured via immunofluorescent staining analysis using specific antibodies. Through flow cytometry, we failed to separate the tumor cells from lymphocytes, as previously reported [41–44]. In tumor sections, treatment with IMQ combined with IR increased the infiltration of CD4^+^ and CD8^+^ T cells in tumor tissues (Figure 5A). In particular, the CD8^+^ T cell number was 2.7-fold higher in the mice treated with IMQ combined with IR compared with the control group. In contrast, IR exposure decreased the number of CD25^+^Foxp3^+^ Treg cells in the tumor sections (Figure 5B). Another set of experiments that aimed to evaluate the Treg population revealed that the number of CD4^+^CD25^+^ cells was also decreased in tumor foci after treatment with IR. This phenomenon was enhanced in the mice treated with IMQ combined with IR. 3-MA treatment alone did not alter the population of T lymphocytes, whereas treatment with IR in combination with IMQ significantly abolished the accumulation of CD8^+^ T cells and the decrease in Tregs in tumor lesions. Additionally, the increased number of CD8^+^ T cells and decreased number of Tregs produced an 18.7-fold elevation in the CD8^+^ T cell to Treg ratio in the mice treated with IMQ and IR compared with the non-treated mice. These alterations in T cell populations might correlate with the inhibition of tumor growth in mice.

MDSCs have been described as an extremely heterogeneous population of immature myeloid cells representing precursors of granulocytes, macrophages, and DCs [45, 46]. The number of CD11b^+^Gr1^+^ MDSCs in tumor lesions was significantly decreased in the mice treated with IMQ combined with IR compared with the untreated mice (Figure 5C). However, treatment with 3-MA after IMQ and IR pretreatment restored the frequency of MDSCs to the control levels. Therefore, an enhanced anti-cancer immune response in tumor foci was confirmed based on the simultaneous increase in the number of CD8^+^ T cells and decrease in the numbers of Tregs and MDSCs. These effects support the inhibition of tumor growth in melanoma-bearing mice.

### Synergistic anti-metastatic effect of IMQ combined with IR

**Figure 6:**
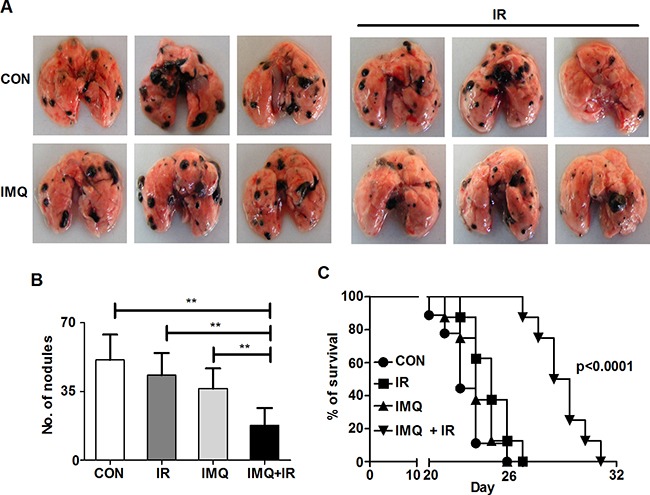
IMQ and IR exert synergistic anti-metastatic effects **A**. Photographs of metastatic nodules in each mouse lung. **B**. The metastatic nodules in the lungs of C57BL/6 mice (n=4/group) treated with saline (CON), IR alone (IR), IMQ alone (IMQ) or IMQ combined with IR (IMQ+IR) for 14 days after B16F10 cell injection into the tail vain were counted. All of the experiments were independently repeated three times for each condition with similar results. Significant differences are indicated by **p < 0.01. **C**. Survival time of four groups of mice (n=8/group). A significant difference p < 0.0001 was found for the comparison of the CON with IMQ+IR groups based on a Kaplan-Meier analysis.

It has been reported that after melanoma metastasizes to distant organs, the cancer becomes more resistant to radiotherapy [47]. This study aimed to determine whether combined treatment with IMQ and IR effectively suppresses melanoma metastasis in a mouse model. It is well known that intravenously transferred B16F10 cells predominantly migrate to and rapidly replicate in the lungs [48]. In this lung metastatic model, the number of black nodules in the lungs of each group was counted, and the survival duration was assessed. IMQ combined with IR reduced the number of metastatic nodules and extended the survival duration (Figures 6A, 6B and 6C). The number of metastatic nodules at 14 days after inoculation was reduced by up to 250% in mice treated with IMQ and IR relative to untreated mice (Figure 6A and 6B). The median survival duration of the control group was 22 days, and that of the group treated with IMQ combined with IR was extended to 28.5 days (Figure 6C). Therefore, IMQ combined with IR increased the survival duration in metastatic melanoma model mice.

### IMQ treatment enhances the anti-cancer and anti-metastatic activities of systemic immunity during radiotherapy for melanoma

**Figure 7:**
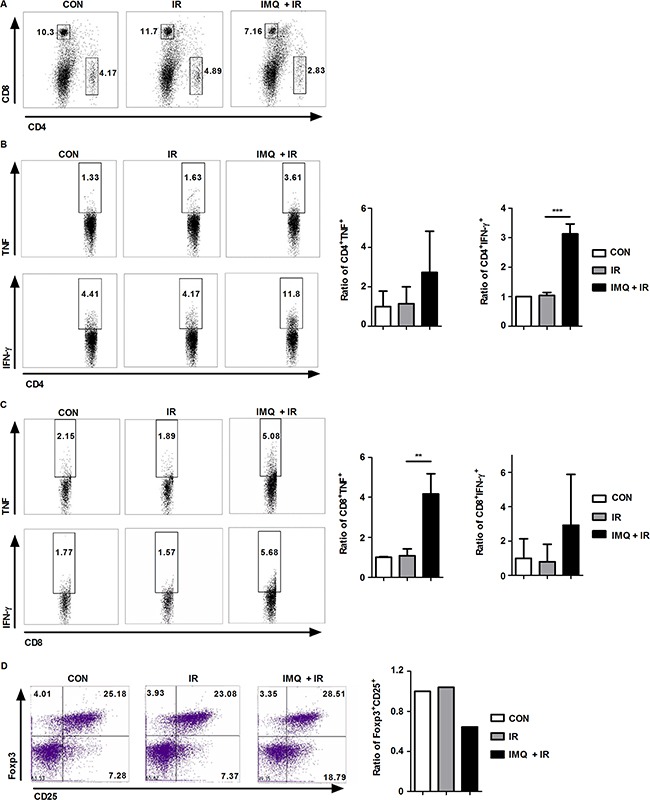
IMQ combined with IR enhances systemic anti-cancer immunity **A**. Splenocytes from each group were isolated and stained with anti-CD4 and anti-CD8 antibodies. **B**. TNF-α- or IFN-γ-producing CD4^+^ T cells are shown. **C**. TNF-α- or IFN-γ-producing CD8^+^ T cells are shown. **D**. Treg cell population in each group. Similar results were obtained from two separate experiments. The graphs in the right panel present the proportions of cells in each experimental group relative to the percentage of cells in the control group. Significant differences are indicated by *p < 0.05, **p < 0.01, and ***p < 0.001.

To investigate whether IMQ reduces the metastasis of melanoma to the lung by enhancing systemic immunity, splenocytes were isolated from the groups of mice, and intracellular cytokine staining was then performed on day 14 using specific cell-surface markers (Figure 7). Although the total numbers of CD4^+^ and CD8^+^ T cells were reduced in the groups treated with IR alone or with both IMQ and IR, the proportions of IFN-γ- or TNF-α-producing CD4^+^ and CD8^+^ T cell populations were notably increased in the spleens of mice simultaneously treated with IMQ and IR (Figure 7B and 7C). Additionally, significant increases in the proportions of IFN-γ-producing CD4^+^ T cells and TNF-α-producing CD8^+^ T cells were detected in the IMQ combined with IR group. Conversely, the Treg frequency was decreased in the group treated with IMQ combined with IR compared with the untreated group (Figure 7D). Therefore, the systemic anti-cancer immune response was enhanced by the combined treatment with IMQ and IR in mice, and this enhancement might be responsible for the reduced metastatic activity of circulating B16F10 cells.

## DISCUSSION

Conventional cancer therapy primarily aims to locally destroy cancer by promoting DNA damage in cancer cells. Apoptosis is a well known programmed cell death mechanism and the primary target of cancer chemo- or radio therapy [49]. A few recent studies suggested that autophagy represents another cell death mechanism that is activated in response to certain stimuli; thus, autophagic cell death is referred to as programmed cell death II [50]. Autophagy could be a survival strategy for cancer cells that are experiencing nutritional starvation under hypoxic conditions [51]. Alternatively, excessively stressful stimuli could promote cell death via macroautophagy. Furthermore, it has been reported that autophagy could enhance the radiosensitivity of apoptosis-resistant cancer cells [52]. Therefore, autophagic signaling could disturb the balance between cellular survival and death.

Although many solid tumors express endosomal TLRs [53], the molecular and cellular function of tumor cell-expressing TLRs in tumorigenesis and metastasis are currently unclear. We previously evaluated the ability of endosomal TLR agonists, including poly(I:C) for TLR3 and IMQ, doxorubicin, and gardiquimod for TLR7, to cause autophagy in MCF-7 human breast cancer cells, and found that poly(I:C) and IMQ increased autophagy-induced cell death in response to low-dose IR exposure [13]. The present study shows that the TLR7 agonist IMQ promotes autophagic cell death in melanoma cell lines and enhances radiosensitivity in an *in vivo* mouse melanoma model, and these effects are correlated with the anti-cancer immune response in tumor lesions. It is known that the inhibition of autophagy could lead to another type of cell death, such as apoptosis [19]. However, 3-MA treatment did not reduce the tumor volume in the control group and 3-MA treatment accelerated tumor growth in the IMQ or IMQ+ IR group in our system. Moreover, IMQ combined with IR is found to decrease the efficiency of melanoma metastasis to the lung and prolong the survival duration of mice by promoting systemic anti-cancer immunity.

It was previously reported that the immune cell component is predictive of a good response to chemotherapy and that increased T lymphocyte infiltration is associated with a higher response rate of breast cancer to therapy [54]. A recent study showed that tumors containing high numbers of suppressor cells and low numbers of cytotoxic T cells respond poorly to chemotherapy and that the survival of mammary tumor-bearing mice is extended via CD8^+^ T cell-influencing mechanisms [55]. Moreover, radiation has been associated with increased expression of the effector cytokines IFN-γ and TNF-α by CD4^+^ and CD8^+^ T cells [56]. Among the T cell populations, Tregs are key contributors to the suppression of tumor-induced immune responses, and this characteristic renders these cells one of the most attractive targets of cancer therapy [57]. According to our results, combined therapy with IMQ and IR promotes CD8^+^ T cell infiltration and suppresses Treg infiltration in tumor lesions, reflecting an enhancement of anti-cancer immunity. The available experimental evidence shows that an elevated number of MDSCs are correlated with therapy failure and poor prognosis in cancer animal models and in cancer patients [58]. Significantly elevated levels of MDSCs have also been detected in melanoma lesions as well as in the spleen and bone marrow [59]. In the present study, enhanced anti-tumor immunity was observed in tumor lesions of tumor-bearing mice treated with IMQ combined with IR, and this conclusion was supported by increased frequencies of CD4^+^ and CD8^+^ T cells in association with reduced frequencies of Treg and MDSC populations in tumor foci.

Despite the anti-cancer and radiosensitizing effects of IMQ, its anti-metastatic activity is largely unknown. Given that metastasized melanoma is a fatal disease in most cases [60], we introduced a metastatic melanoma animal model and treated these mice with IMQ once before IR exposure and again after IR exposure. A recent study demonstrated that the TLR2/6 agonist MALP-2 prevents the metastasis of melanoma to the lungs [61]. However, whether other TLR agonists exert anti-metastatic effects during radiotherapy has not yet been elucidated. The current data provide the first evidence that IMQ has the potential to suppress metastasis in a murine melanoma model via a TLR7-induced pathway. Additionally, the present results strongly indicate that IMQ increases protective systemic immunity *in vivo* and exerts a direct cytotoxic effect on tumor cells.

TLR agonists have been shown to activate the host immune system as adjuvants and have emerged as a new class of anti-cancer vaccine booster. The administration of TLR agonists to a tumor-bearing animal model or melanoma patient promotes radiosensitization and activates innate immune responses by stimulating DC maturation [62], inflammatory responses and costimulatory molecules that assist in antigen presentation. In addition, enhancing the immune response could help the host eradicate cancer. Several studies have shown that stimulating the TLR pathway enhances cytotoxic T cell activity and stimulates NK cells to kill cancer cells [63, 64]. Therefore, treatment with IMQ as an anti-melanoma drug to promote autophagy in cancer cells and to further sensitize these cells to radio- or chemo-therapy could lead to specific immune responses that fight against cancer. These findings also indicated that IMQ could be investigated as a candidate adjuvant to radiotherapy for the treatment of radio-resistant melanoma.

## MATERIALS AND METHODS

### Cell lines

B16F1 and B16F10 mouse melanoma cell lines were originally purchased from the American Type Culture Collection (Manassas, VA, USA) and were maintained in DMEM containing 10% of fetal bovine serum (FBS; Hyclone Laboratories, Logan, UT, USA) supplemented with 1,000,000 U/L penicillin and 100 mg/L streptomycin (Gibco-BRL, Gaithersburg, MD, USA).

### Reagents

IMQ was purchased from InvivoGen (SanDiego, CA, USA). 3-MA was purchased from Sigma-Aldrich (St. Louis, MO, USA). For western blot analysis, anti-β-actin, anti-LC3B, anti-Beclin-1 and anti-Atg5-12 antibodies were purchased from Cell Signaling Technologies (Danvers, MA, USA). Anti-phospho-Erk1/2, anti-Erk1/2, anti-phospho-p38, anti-p38 and anti-phospho-IκBα were purchased from Cell Signaling Technologies (Danvers, MA, USA). Anti-phospho-NF-κB p65, anti-NF-κB p65 and β-actin were purchased from Santa Cruz Biotechnology (Dallas, TX, USA). The other reagent sources were as follows: diphenyleneiodonium chloride (DPI) (Calbiochem, USA), apocynin (Sigma-Aldrich, USA), and DCFH-DA (Calbiochem, USA). Anti-CD4-FITC, anti-CD8-FITC, anti-CD11b-FITC, anti-FOXP3-Alexa 488 (126406), anti-Ly6C-APC, anti-CD45 (103124), anti-Ly6G-PE/Cy7 (15-5931-82), anti-TNFα-APC (17-7321-82), anti-IFN-γ-APC (17-7311-82), anti-Gr-1-Alexa 488 (108417) and anti-CD25-Alexa 647 (102020) antibodies were purchased from eBioscience (San Diego, CA, USA). For immunostaining, an anti-LC3 antibody was purchased from MBL International Corporation (Nagoya, Japan), and a FITC-conjugated anti-rabbit secondary antibody was purchased from Jackson Immuno Research (West Grove, PA, USA).

### Radiation treatment

Cells and mice were subjected to whole-body IR exposure (2 Gy; dose rate of 0.6 Gy/min) using a Gamma Cell Cesium-137 unit (Gammacell 3000 Elan, Best Theratronics, Ottawa, ON, Canada) at the Korea Institute of Radiological and Medical Sciences.

### Tumor formation and treatment with IMQ and 3-MA

Male 6- to 8 week-old C57BL/6 mice were obtained from Nara Biotech (Pyeong-taek, Korea). All experimental animals used in this study were handled using a protocol approved by the Institutional Animal Care and Use Committee of Kangwon National University (Permit Number: KW-130613-2). A total of 5 × 10^5^ B16F1 or B16F10 cells were injected into the hind limb of each mouse. The tumors were allowed to grow for 10 days and the tumor volume was measured every day using skin calipers and calculated according to the following formula: width^2^ x length x 0.5. One hundred microliters of IMQ (10 μg/mL) were subcutaneously injected near the tumor foci twice: one dose was administered 6 h prior to IR exposure, and the second dose was administered on the day after IR. 3-MA at a concentration of 2 mg/kg was injected near the tumor foci every day from the day prior to IR exposure until the day that the mice were scarified. The tumor sections were subjected to western blot and histology analyses. The mice were killed 21 days after melanoma cell inoculation.

### Cell proliferation assay

Cell proliferation assays were performed using WST-1 (Takara, Otsu, Japan) according to the manufacturer's instruction. Briefly, B16F1 or B16F10 cells (1 × 10^4^ per well) were plated in 96-well microplates for one day and then treated with IMQ after pretreatment with 3-MA (10 mM). At each time point, the WST-1 reagent was added to the 96-well plates, and the plates were then incubated for 4 h. The absorbances at 450 nm and/or 690 nm were measured using a plate reader (Biotek Instruments Inc., Winoski, VT, USA).

### Western blot analysis

Cells were harvested after the indicated treatment and cell lysates were prepared for western blotting analysis as previously described [13]. Briefly, proteins were separated via 10-15% SDS polyacrylamide gel electrophoresis, transferred to a PVDF membrane (Millipore, Bedford, MA, USA) and then subjected to western blot using the indicated antibodies. The membrane was treated with ECL (Animal Genetics Inc., Gyeonggi-do, Korea) and exposed to medical X-ray film (AGFA, Mortsel, Belgium) in a darkroom. The housekeeping protein β-actin was used to confirm equal loading of the wells.

### RT-PCR analysis

Total cellular RNA was extracted using the RNeasy Mini Kit (Qiagen, Valencia, CA, USA) according to the manufacturer's instruction. cDNA synthesis and RT-PCR analysis were performed as previously described [65]. Briefly, cDNA was synthesized using M-MLV reverse transcriptase (Takara, Japan) in a reaction including RNase inhibitors and a random hexamer (Takara, Japan); and was then subjected to PCR for amplification. The sets of primer sequences used for amplifying *tlr7* were 5‘-GGC TAC CAG GAC AGC CAG TTC-3′ and 5‘-GCC ACA TGA TTG TCT GTG GTC-3′. The sequences of the *beta-actin* primers were 5′- AGG CTG TGC TGT CCC TGT ATG C-3′ and 5′- ACC CAA GAA GGA AGG CTG GAA A-3′. β-actin was used as an internal control. The amplified products were separated on a 1.5% agarose gel and visualized after staining with ethidium bromide (Sigma-Aldrich, St. Louis, MO, USA).

### ROS measurement

Intracellular ROS was determined using a fluorescence-based dye, 2′,7′-dichlorofluoresecin diacetate (DCFH-DA, Calbiochem, USA), as previously described [66]. Briefly, cells were plated on coverslips in 12-well plates, pre-treated with DPI or apocynin for 1 h washed with PBS and treated with IMQ alone or in combination with IR. The cells were treated with complete medium containing DCFH-DA for 20 min at 37°C in 5% CO_2_. And then, cells were washed twice with PBS and fixed in PBS containing 4% paraformaldehyde. The coverslips were mounted in Fluoromount-G^TM^ (Southern Biotech, Birmingham, USA) and examined using a confocal microscope (FV1000 SPD, Olympus, Tokyo, Japan).

### Immunofluorescence staining

Cells were seeded on 12-well culture plates that contained 18 mm-diameter round glass coverslips and treated with IMQ or IR. Briefly, the cells were washed with PBS and fixed with PBS containing 4% paraformaldehyde for 10 min. After fixation, the cells were lysed in 0.2% Triton X-100 for 10 min, incubated with an anti-LC3 antibody (MBL, Nagoya, Japan) for 2 h, and then incubated with a FITC-conjugated anti-rabbit secondary antibody (Jackson Immuno Research, West Grove, PA, USA) for 1 h at room temperature. The nuclei were stained with 4′-6-diamidino-2-phenyl indole (DAPI, Sigma-Aldrich, St. Louis, MO, USA) for 5 min [13].

Tumor tissue was collected from C57BL/6 mice and fixed in 10% formalin. Cryostat sections (5-10 μm in thickness) were embedded in frozen FSC22 (Surgipath, Richmond, IL, USA) on dry ice and then, air-dried for 1 h. For immunostaining, the tumor sections were incubated with antibodies against CD8, CD11b, Gr-1, Foxp3, CD25 and LC3 for 2 h. After this incubation, the sections were washed twice with PBS and then stained with DAPI for nuclear staining. All images were examined using a confocal laser scanning microscope (FV1000 SPD, Olympus, Tokyo, Japan).

### Cell isolation

The mice were sacrificed under deep anesthesia, and tumor tissues and spleens were collected. For splenocyte collection, the spleens were removed from C57BL/6 mice through an abdominal incision, and each spleen was then cut into approximately three to four pieces with sterile scissors on a sterile culture dish containing RPMI-1640 media. The spleen pieces were gently minced and passed through a 40-μm sterile cell strainer (SPL Life Sciences, Pocheon, Korea) to remove the dispersed cells from the tissue fragments. After centrifugation, the RBCs were removed from the collected cells using RBC lysis buffer. For immune cell subset isolation, the tumor tissue from C57BL/6 mice was collected, minced and passed through a cell strainer. Cell suspensions were layered over Ficoll-Paque PLUS (GE Healthcare Bio-Science, NJ, USA) and centrifuged at 1,500 rpm. The purified splenocytes and immune cell subpopulations were used for flow cytometry.

### Flow cytometry

The cells were pelleted via centrifugation and treated with 1X RBC lysis buffer (8.26 g of ammonium chloride (NH_4_Cl), 1 g of potassium bicarbonate (KHCO_3_) and 0.037 g of EDTA dissolved in 1 L of H_2_O) to lyse erythrocytes. The lysed erythrocytes were removed by washing twice with RPMI-1640 medium at 1000 rpm for 10 min. The cells were aliquoted based on the experimental requirements and used for all further cell-based assays. The cells were rinsed with 1X PBS and fixed with 1% paraformaldehyde in PBS for 15 min at 4°C. The fixed cells were washed with PBS and suspended in chilled FACS buffer (1X PBS containing 1% FBS and 0.02% sodium azide) at a density of 1×10^6^ cells/500 μL of buffer in each tube. For surface marker analysis via multicolor immunophenotyping, the non-permeabilized cells were stained with anti-CD4-FITC, anti-CD8-FITC, anti-CD11b-FITC, anti-FOXP3-Alexa 488 (126406), anti-Ly6C-APC, anti-CD45 (103124), anti-Ly6G-PE/Cy7 (15-5931-82), anti-Gr-1-Alexa 488 (108417) and anti-CD25-Alexa 647 (102020) antibodies at 4°C. For intracellular staining, the fixed cells were washed with PBS and permeabilized for 15 min at room temperature in saponin buffer (FACS buffer containing 0.1% saponin). The permeabilized cells were washed with saponin buffer, suspended in FACS buffer at a density of 1×10^6^ cells/50 μL of buffer in each tube and stained with anti-TNFα-APC (17-7321-82) and anti-IFN-γ-APC (17-7311-82) antibodies for 30 min at room temperature. The appropriate isotype controls were used in all experiments. After the incubation period, the cells were washed via centrifugation using FACS buffer, and the pellet was suspended in 500 μL of FACS buffer. Cells were acquired using a FACSVerse flow cytometer (BD Bioscience), and the data were analyzed using BD FACSuite software.

### Metastatic animal model

A total of 1×10^6^ B16F10 cells were administered via intravenous injection into the tail vein. The mice were sacrificed 14 days after injection. One hundred microliters of IMQ (10 μg/mL) were intraperitoneally injected twice: one dose was administered 6 h prior to IR exposure, and the second dose was administered the day following IR. The number of lung tumor nodules on the tissue surface was counted, and the percentage of lung tumor nodules relative to the number of lung tumor nodules in untreated tumor-bearing mice was calculated. The survival of the mice was subsequently monitored daily for a period of 31 days.

### Histology

After measuring the tumor volume, the tumor tissues were fixed with 10% formaldehyde for 24 h and embedded in paraffin. Sections (2 μm) were stained with hematoxylin and eosin. The hematoxylin and eosin stain was purchased from Sigma-Aldrich (St. Louis, MO, USA).

### Densitometric analysis and statistics

The experiments were repeated at least three times to ensure consistent results. The significance levels used for comparisons between samples were determined using Student's t test or ANOVA with GraphPad Prism 5 software (GraphPad Software, San Diego, CA, USA). Statistical significance was indicated by a *p* value of less than 0.05 (*p < 0.01, ^*^p < 0.05, ^**^*p < 0.001). The Kaplan-Meier method was used to determine the statistical significance of the differences in the survival duration between groups.

## SUPPLEMENTARY MATERIALS FIGURES


