# Supplementary material for: The TLR7 agonist imiquimod induces anti-cancer effects via autophagic cell death and enhances anti-tumoral and systemic immunity during radiotherapy for melanoma
Source: Oncotarget. 2017 Feb 15;8(15):24932–48. doi: 10.18632/oncotarget.15326 (PMC5421900; doi:10.18632/oncotarget.15326)
Supplement: Supplementary file 1 [file oncotarget-08-24932-s001.pdf]

The TLR7 agonist imiquimod induces anti-cancer effects via autophagic cell death and enhances anti-tumoral and systemic immunity during radiotherapy for melanoma

SUPPLEMENTARY FIGURES

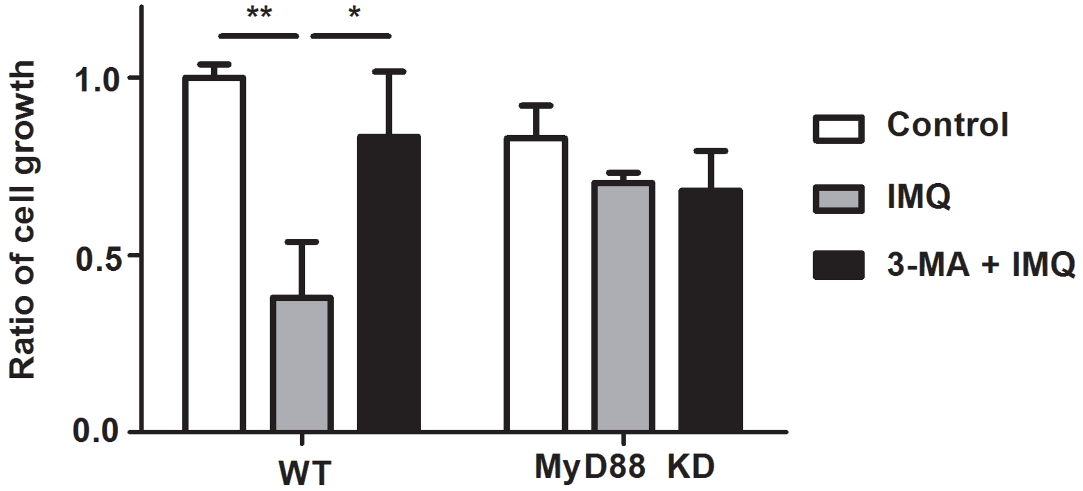

Supplementary Figure 1: Cytotoxic effect of IMQ is dependent on TLR7 pathway. The growth rates of untreated cells (Control), IMQ-treated cells (IMQ), and 3-MA and IMQ-treated cells (3MA+IMQ) were measured in both wild-type and Myd88 knock-down B16F10 cells.

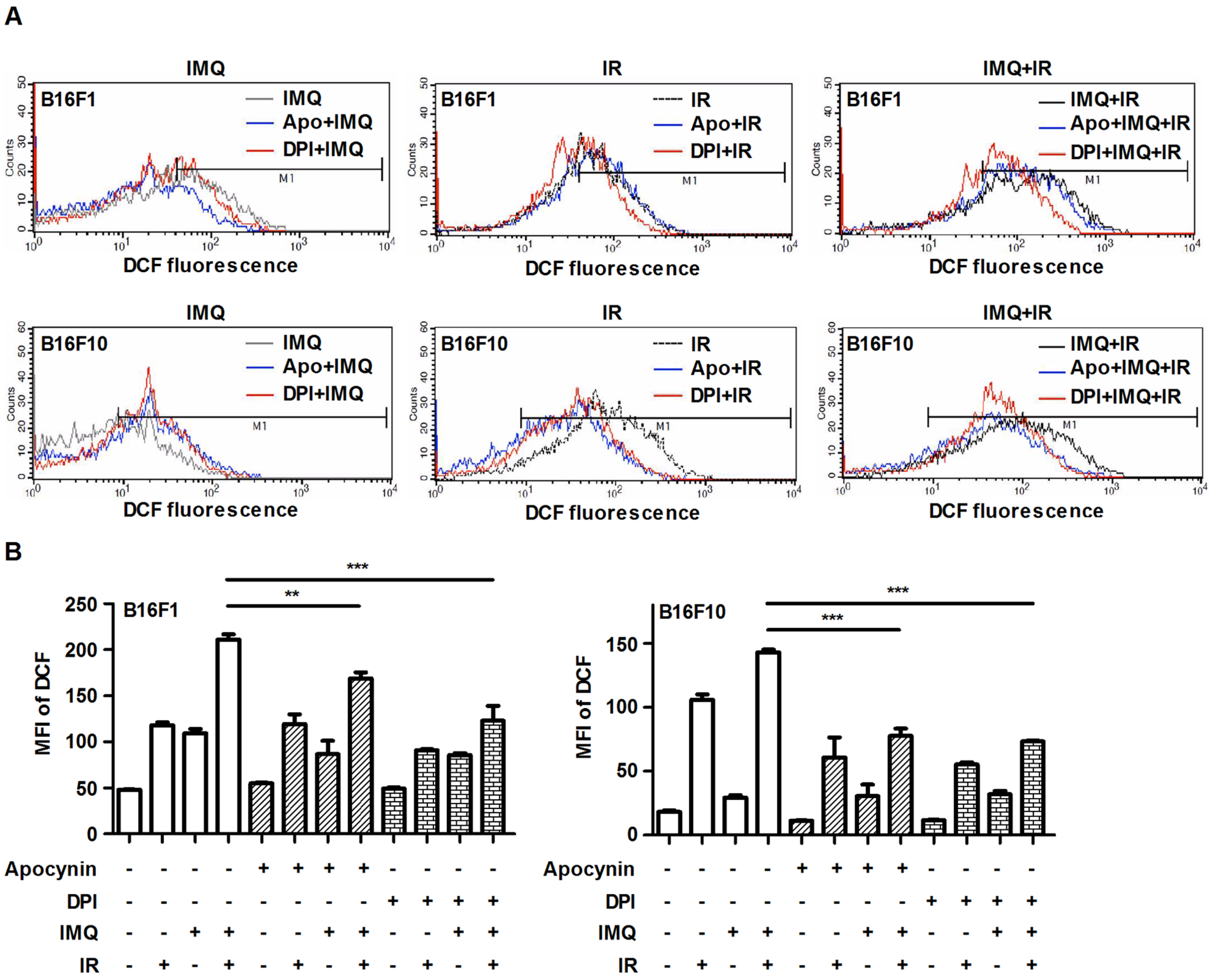

Supplementary Figure 2: IMQ treatment combined IR enhances ROS production. **(A)** B16F1 and B16F10 cells were treated with IR alone (IR), IMQ alone (IMQ) or IMQ combined with IR (IMQ+IR) in the presence or absence of ROS scavenger, DPI or NADPH oxidase inhibitor, apocynin (Apo). Cells were labeled with 5-[and-6]- chloromethyl-2', 7'-dichlorodihydro fluorescein diacetate (DCFH-DA) and then evaluated using flow cytometry. **(B)** The bar graphs represent DCF MFI. The mean value ± standard deviation from triplicate measurements are shown. The MFI refers to the mean fluorescence intensity. Significant differences are indicated by $^{**}p < 0.01$, and $^{***}p < 0.001$.
